# The *E*. *coli* MinCDE system in the regulation of protein patterns and gradients

**DOI:** 10.1007/s00018-019-03218-x

**Published:** 2019-07-17

**Authors:** Beatrice Ramm, Tamara Heermann, Petra Schwille

**Affiliations:** Max Planck Institute of Biochemistry, Am Klopferspitz 18, 82152 Martinsried, Germany

**Keywords:** Reaction–diffusion mechanism, Spatiotemporal regulation, ParA-type ATPase, Geometry sensing, Reconstitution, FtsZ

## Abstract

Molecular self-organziation, also regarded as pattern formation, is crucial for the correct distribution of cellular content. The processes leading to spatiotemporal patterns often involve a multitude of molecules interacting in complex networks, so that only very few cellular pattern-forming systems can be regarded as well understood. Due to its compositional simplicity, the *Escherichia coli* MinCDE system has, thus, become a paradigm for protein pattern formation. This biological reaction diffusion system spatiotemporally positions the division machinery in *E*. *coli* and is closely related to ParA-type ATPases involved in most aspects of spatiotemporal organization in bacteria. The ATPase MinD and the ATPase-activating protein MinE self-organize on the membrane as a reaction matrix. In vivo, these two proteins typically oscillate from pole-to-pole, while in vitro they can form a variety of distinct patterns. MinC is a passenger protein supposedly operating as a downstream cue of the system, coupling it to the division machinery. The MinCDE system has helped to extract not only the principles underlying intracellular patterns, but also how they are shaped by cellular boundaries. Moreover, it serves as a model to investigate how patterns can confer information through specific and non-specific interactions with other molecules. Here, we review how the three Min proteins self-organize to form patterns, their response to geometric boundaries, and how these patterns can in turn induce patterns of other molecules, focusing primarily on experimental approaches and developments.

## Introduction

One of the most intriguing phenomena found in nature is the spontaneous generation of gradients, and thus, of spatial or temporal order, from diffusible entities. This process, often referred to as pattern formation, relies on the ability of the system to dissipate energy, and is a hallmark of biological systems. The concept of pattern formation in biology was initially introduced to describe the translation of genetic information into the spatial organization of differentiating cells [1]. Patterns do not only occur during the segmental organization of developing organisms [1, 2], but can be found across all scales of life: from fish swarm behavior [3] via molecular pathways determining patterning of feather arrays [4] to the organization of intracellular space as in microtubule arrangement [5].

On a cellular level, two fundamentally different mechanisms are responsible for the emergence of spatial organization. The first one, known as molecular self-assembly, describes the physical association of a set of components into a stable structure without energy dissipation, leading to an equilibrium state [6]. However, spatiotemporal order required for life processes crucially depends on a second phenomenon termed self-organization that takes place far from equilibrium. In contrast to self-assembly, self-organization relies on nonlinear and transient interactions between the individual components which consume energy from sources like ATP or GTP [7]. Hence, self-organizing systems acquire emergent properties that cannot be anticipated from the function of the individual subunit [8]. Besides inherent complexity, this allows for an even greater responsiveness enabling, e.g., morphological changes [9].

The micrometer-scale patterns generated by self-organizing protein networks govern various essential cellular processes in both prokaryotes and eukaryotes. Two of the best studied protein-based, self-organizing systems of the eukaryotic cell are the determinants of cell shape and polarity, the actin and microtubule cytoskeletons. In vitro reconstitution of cytoskeletal filaments elegantly demonstrated the underlying self-organizing properties that enable their organization into in vivo-like asters, vortices and spindle networks even in a minimal system [10]. Next to these active systems that consist of cytoskeletal filaments and motor proteins, protein pattern formation can also be based on reaction–diffusion mechanisms. Examples are Cdc42 in *Saccharomyces cerevisiae* [11] or Par proteins in *Caenorhabditis elegans* zygotes [12].

Similar to eukaryotic cells, reaction networks linking self-organization and fundamental functions, such as signal transduction, division or the organization of intracellular space, are also found in prokaryotes. Due to their small size, which allows for efficient space exploration by diffusion, bacteria avail themselves much more of the reaction–diffusion systems. In such systems, as little as two molecules that react with each other in networks of certain topology and have different diffusive properties can give rise to a variety of different patterns from an initially homogenous mixture [13, 14]. As Alan Turing was the first to describe such systems to explain pattern formation in biology in 1952 [13], such patterns have been coined “Turing patterns”. Intriguingly, several of the so-far-discovered nonlinear reaction systems depend on catalytic environments, such as DNA or lipid membranes, to transiently modulate their function. These reaction matrices are especially important in reaction–diffusion systems as they act as modulators of diffusion coefficients or induce conformational changes between active and inactive protein states. A prime example of a reaction–diffusion system self-organizing on lipid membranes is the *Escherichia coli* MinCDE system, which is crucial for the spatiotemporal localization of the division machinery to mid-cell [15].

The *E*. *coli* MinCDE system was first identified by mutations of its corresponding genetic locus *minB* that led to the formation of miniature, anucleate cells, in short: minicells [16, 17]. 30 years ago, de Boer et al. identified the three proteins encoded by *minB*: MinC, MinD and MinE [15]. Gene expression studies of all three genes suggested that MinC and MinD together act as an inhibitor of cell division, while MinE confines this inhibitory activity to the cell poles, operating as a topological specificity factor [15]. While this basic model still holds true to this date, 30 years of research in vivo, in vitro and in silico have revealed the detailed molecular mechanism of the system. Especially, the first in vivo visualization of the fascinating MinCDE pole-to-pole oscillation sparked a flurry of research [18, 19]. Considerably later, the MinDE dynamics were reconstituted in vitro [20], allowing for a controlled and quantitative interrogation of the underlying molecular mechanism and its potential application for the design of minimal cells. Furthermore, the MinCDE system has been subject to extensive mathematical modeling [20–26] to elucidate crucial aspects of the molecular mechanism. We refer the reader to two excellent reviews and a book chapter with focus on the theoretical description of the phenomenon [27–29], as this review will mostly focus on experimental approaches and insights. We will describe the basic mechanism of MinCDE pattern formation, how it results in a variety of patterns in vivo and in vitro, and how these patterns are modulated by geometric constraints. We further outline how MinCDE patterns can induce downstream pattern formation, i.e., the positioning of target molecules. Finally, we give an overview of systems related to the *E*. *coli* MinCDE system and how this system can be applied in synthetic biology and beyond.

## The components of the *E*. *coli* MinCDE system

To understand the molecular mechanism of the MinCDE oscillation cycle, we first need to introduce the individual components: the three proteins MinD, MinE and MinC as well as the reaction matrix, the phospholipid membrane.

### The ATPase MinD

**Fig. 1:**
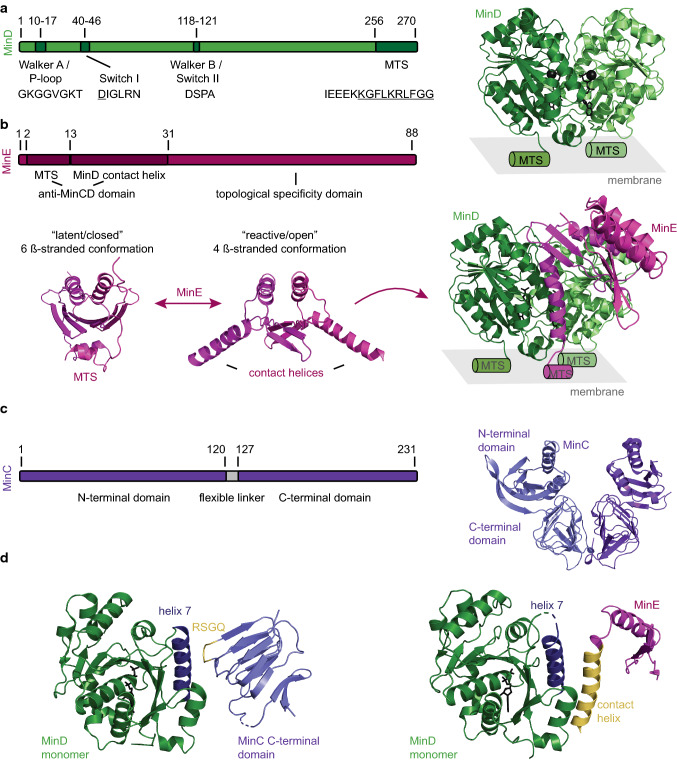
Overview of the three Min proteins. **a** MinD can bind to the membrane via its C-terminal MTS upon ATP-dependent dimerization (residues forming the amphipathic helix are underlined). Schematic view highlighting the structural motifs and their respective amino acid sequences of *E*. *coli* MinD: Walker A and B and switch I motifs required for ATP binding and Mg^2+^ coordination. Crystal structure of the dimeric MinD with ATP and the Mg^2+^ ion shown in black. MTS location is indicated by schematic helices (PDB: 3Q9L [36]). **b** Schematic view highlighting the structural motifs and their respective amino acid sequences of *E*. *coli* MinE. MinE exists in a latent/closed conformation in solution (crystal structure of *Neisseria gonorrhoeae* MinE, PDB:2KX0 [51]). Upon “sensing” MinD on the membrane, it transforms into a reactive/open conformation where the contact helices and MTS are exposed, freeing it to interact with MinD (crystal structure of the *E*. *coli* MinDE complex, note that MinE 13-88 I24N was used, PDB: 3R9J [52]). **c** Schematic view of *E*. *coli* MinC shows that the protein consists of two domains that are connected via a flexible linker. Crystal structure of dimeric MinC from *Thermotoga maritima* (PDB: 1HF2 [53]). **d** MinC and MinE have overlapping binding interfaces on MinD. Crystal structure of the *Aquifex aeolicus* MinD monomer with helix 7 highlighted in blue and the C-terminal domain of *A*. *aeolicus* MinC with the RSGQ motif displayed in yellow (left, PDB: 4V02 [54], note that helix 3 of *A*. *aeolicus* is not shown as it is absent in MinC of most bacterial species including *E*. *coli* [55]). Crystal structure of the *E*. *coli* MinD monomer with helix 7 highlighted in blue and MinE with the contact helix displayed in yellow (right, PDB: 3R9J [52], note that only the monomer of MinE is shown)

MinD is a dimeric ATPase that belongs to the P-loop (phosphate binding loop) NTPases of the SIMIBI class (signal recognition particle, MinD, BioD) [30]. Some members of this family, MinD as well as nucleoid-guided ParA ATPases, have also been termed Walker A cytoskeletal ATPases [31]. They are characterized by a conserved N-terminal Walker A motif or P-loop (*E*. *coli* MinD: AA 10–17, GKGGVGKT) and the more central Walker B motif or switch II region (*E*. *coli* MinD: AA 118–121, DSPA), that coordinate the triphosphate group of the ATP and complex the Mg^2+^ ion, respectively (Fig. 1a) [32–35]. A third motif that participates in nucleotide binding is termed switch I region and harbors a conserved aspartate that probably coordinates the attacking, nucleophilic water molecule during ATP hydrolysis (*E*. *coli* MinD: AA 40–46, DIGLRN) [33, 34, 36]. In G proteins, the switch I and II residues undergo nucleotide-dependent conformational changes mediating interactions. Similarly, in MinD, these motifs seem to mediate binding and activation of MinC [37]. The Walker A motif of MinD deviates from the classical motif in that it contains a signature lysine (*E*. *coli* MinD, K11) [30]. This lysine interacts with an aspartate (*E*. *coli* MinD, D152) in the monomeric, ADP-bound state, but this interaction is abrogated when the protein binds ATP: the protein dimerizes and the signature lysine then contacts the ATP of the other monomer [36, 38]. At the far C-terminus, MinD harbors an amphipathic helix also termed membrane-targeting sequence (MTS) (*E*. *coli* MinD: AA 256–270) [39, 40]. This amphipathic helix has a rather weak membrane affinity and supports membrane binding only at higher valencies, i.e., when two or more copies of the MTS are present [41, 42]. Hence, the nucleotide state determines the localization of MinD acting as a molecular switch: in its ADP-bound state, the protein is monomeric and soluble, but dimerizes upon exchanging the nucleotide for ATP which in turn enables membrane binding [32, 33, 36, 43, 44] (Fig. 1a). MinD binds to the membrane as a monomolecular layer of about 5 nm height [45] and the binding process is highly cooperative [44–46]. Most mathematical models reproducing MinDE oscillations either require MinD filament formation or incorporate a not further specified “MinD recruitment”, where more MinD on the membrane recruits additional protein from the cytosol [23, 24, 28]. The observed cooperativity can only partly be explained by the ATP-dependent dimerization that allows for membrane binding. There have been several reports of MinD forming filamentous structures in the presence of phospholipids that could be disassembled by MinE [43, 47]. However, these filaments could only be observed at high protein concentrations [43, 47]. A recent high-speed atomic force microscopy study visualized MinDE dynamics at high spatial and temporal resolution: MinDE formed higher-order structures that rapidly diffuse and disassemble on the membrane, resembling a 2D crystal-like, but highly dynamic packing of MinD dimers [45]. Another study supports the formation of higher-order MinD structures on the membrane, as MinDE waves can displace fluorescent proteins with the same membrane affinity as a MinD dimer (mCherry fusion to two *E*. *coli* MinD MTS) [42]. Indeed when the MinD density on the membrane is low in vitro, individual MinD dimers are rapidly diffusing on the membrane and have a short membrane residence time [45, 48]. In contrast, increasing MinD densities reduce lateral diffusion and increase membrane residence time, thus supporting the assumption of lateral interactions between MinD dimers [45, 48]. Hence, for efficient membrane detachment, MinD needs to return to the monomeric, ADP-bound state by ATP hydrolysis. MinD itself possesses a low intrinsic ATPase activity [44, 49, 50]. However, when MinD is membrane bound, this activity can be stimulated by MinE, enabling monomerization and membrane detachment [44, 50].

### The ATPase-activating protein MinE

MinE is the cognate ATPase-activating protein of MinD. It is a small protein of only 88 amino acids that is divided into two functional domains: the anti-MinCD domain (*E*. *coli*, AA 1–31), sufficient to suppress MinCD inhibitory activity in vivo, and the topological specificity domain (*E*. *coli*, TSD, AA 32–88) that antagonizes the inhibitory activity of the former only at midcell, restricting its activity to the cell poles [56–58] (Fig. 1b). The anti-MinCD domain further consists of two functional motifs: an N-terminal membrane-targeting sequence (MTS) spanning residues 2–12 (*E*. *coli* MinE) [59, 60], and the MinD contact helix located between amino acids 13 and 31 (*E*. *coli* MinE) [52, 61] (Fig. 1b).

The TSD is in fact a dimerization domain that promotes the formation of MinE homodimers. Genetic studies with MinE mutants inducing minicell formation when expressed in *E*. *coli* as well as yeast two-hybrid analysis suggested strong self-interaction properties of the TSD [56, 57]. Indeed, MinE was shown to self-associate in vivo: simultaneous expression of wild-type and MinE 22–88 led to the formation of heterodimers, and thus the inhibition of cell division [62]. Shortly thereafter, the structure of the constitutive MinE dimer was released [58].

Similar to MinD, MinE possesses a membrane-targeting sequence (MTS) which in contrast to MinD is located at the N-terminus (*E*. *coli*, residues 2–12) [59, 60]. Biochemical characterization of this motif suggests that it is composed of an amphipathic helix, where the hydrophobic residues A2, L4 and F6 insert into the lipid bilayer, and a sequence of positively charged amino acids R10, K11 and K12 electrostatically interact with the negatively charged phospholipid headgroups (referring to *E*. *coli* MinE) [59, 60]. To ensure a weak affinity for the *E*. *coli* membrane in the absence of MinD, the MTS and the neighboring contact helix are concealed in the hydrophobic core of the MinE dimer, through interaction with the TSD [51, 52, 59] (Fig. 1b). Direct membrane interaction is in principle not crucial for MinE to antagonize MinD [24] and MinE mutants lacking the MTS support pattern formation in vitro, but with altered length-scale and dynamics [63, 64]. However, several in vivo, in vitro and theoretical studies demonstrated the necessity of the direct MinE–membrane interaction for functional MinCDE oscillations in vivo [36, 48, 52, 59, 63–65].

The contact helix spanning amino acid positions 13–31 (*E*. *coli* MinE), is crucial for the MinE–MinD interaction and confers the primary function of MinE to activate the ATPase activity of MinD in the presence of phospholipid membranes [50, 61]. Intriguingly, these residues only fold into an α-helix upon interaction with MinD [61]. MinE dimers switch between a closed/latent state, composed of a 6-stranded, anti-parallel β-sheet, and an open/active, 4ß-stranded conformation in complex with MinD [52] (Fig. 1b). The latent state, in which also the MTS is sequestered, is able to freely diffuse in the cytoplasm until the MinE dimer encounters an ATP-bound MinD dimer on the membrane [66, 67]. Upon interaction, MinE transforms into its open (4ß-strand) conformation through a multistep process, which releases the MTS and the contact helix [66, 67].

The resulting MinDE complex is asymmetric, as the MinE dimer is bound on only one side of the MinD dimer [68]. In this complex, MinE stimulates ATPase activity of MinD presumably by inducing a conformational change in the *E*. *coli* MinD switch I residue N45 [44, 50, 68]. Despite the asymmetry, MinE binding triggers ATP hydrolysis in both MinD subunits, which induces MinD monomerization and membrane detachment (Fig. 1b) [68].

After MinD has dissociated from the membrane, several studies showed that MinE dimers “linger” bound to the lipid bilayer, thus directing the de novo binding of MinD to the opposing cell pole [48, 59, 69]. It was initially unclear why MinE features a conformational switch rather than remaining in its active form, but it was recently shown that the MinE switch confers robustness to the Min oscillations sustaining pattern formation over a wider concentration range [25, 64].

Despite our rather detailed knowledge about the structure and the conformational freedom of MinE, the mode of action in vivo remains unclear. Several different, but partly congruent mechanisms have been proposed [21, 24, 25, 70, 71]. One model has been termed the “Tarzan of the jungle”, in the way that MinE acts as Tarzan moving ‘hand over hand’ from one membrane-bound MinD to the other as if they were “vines” [52]. If lacking a binding partner in close proximity, MinE dissociates from the membrane. Thus, the fate of MinE is determined by the local density of MinD, which either ensures successful rebinding or the return to the inactive, 6-ß-stranded conformation [52]. Although it was also assumed that there might be an intermediate state in which MinE is transiently bound to the membrane without MinD, this model does not emphasize free, membrane-bound MinE. Another model explains the acceleration of protein detachment at the rear of the Min wave through a MinE-induced positive feedback [48, 72]. This feedback originates from MinE ability to perform two complementary actions: rapid rebinding and persistent membrane binding [48]. A proposed alternative is the so-called MinDE toggle switch, which assumes that the local stoichiometry of MinDE on the membrane either promotes MinE-stimulated MinD recruitment (MinD excess state) or the MinE-stimulated MinD release (MinE excess state) [63]. That implies that the membrane-bound MinD is stabilized by MinE, recruits further MinD until MinE tries to balance the concentration gradient and the MinDE complex is able to bind to another MinE, thus inducing the dissociation of the complex formed by two MinE and a MinD complex from the membrane [63].

### The effector protein MinC

MinC is not participating in the MinDE dynamics, but can be seen as the effector of the system. As an inhibitor of FtsZ assembly, it confers the ability of the MinCDE system to inhibit and position cell division (see below). MinC is a dimeric protein, where each monomer further consists of two domains connected by a flexible linker, which allows free rotation of the N-terminal domains [53, 73] (Fig. 1c). Both the N-terminal and C-terminal domain harbor FtsZ inhibitory activity [74–76]. The C-terminal domain contains the dimerization interface consisting of primarily hydrophobic residues [53, 54, 73]. It further comprises the conserved residues, RSGQ (in *E*. *coli* MinC), that mediate the interaction with MinD [38, 54]. Dimeric, ATP-bound MinD binds MinC and can thereby recruit MinC to the membrane in vitro and in vivo [19, 44, 52, 77, 78]. MinC and MinE binding sites on the MinD surface are overlapping [36, 55, 79]. Specifically, the RSGQ motif of MinC as well as the contact helix of MinE interact with residues S148, D154 and I159 in helix 7 of MinD (referring to the *E*. *coli* MinCDE system) [36, 79] (Fig. 1d). Thus, competition for the same binding site enables MinE to displace MinC from membrane-bound MinD [44, 77]. This shared binding site presumably also explains how MinC can interfere with MinDE pattern formation in vitro: unusually high MinC concentrations can disturb MinDE pattern formation in vitro, presumably by outcompeting MinE binding to MinD [80].

### The lipid membrane as a reaction platform

One of the most crucial determinants for MinDE pattern formation is the ability of MinDE to interact with the phospholipid bilayer interface via their MTS [41, 43, 59, 60]. Due to this process, the diffusion constants of the proteins can be significantly decreased, and in combination with molecular interactions, enable the dynamic instability needed for self-organizing patterns [20, 48]. Remarkably, the membrane can, thus, be considered as a heterogeneous catalyst for pattern formation, in the same way as platinum substrates for CO oxidation [81]. For the interaction with the membrane, MinDE oscillations require a specific anionic charge density of the membrane, reflecting the nature of their positively charged MTS [46, 69, 82]. Interestingly, anionic lipids such as cardiolipin were shown to be concentrated at the cell poles, and when cells are stained with dyes specific for anionic phospholipids such as cardiolipin, the resulting densities resemble MinD localization at the cell poles and nascent septa [83, 84]. These findings led to the suggestion that the distribution of certain lipids acts as a spatial cue for MinDE localization. However, it has been demonstrated that only the net negative charge, rather than cardiolipin itself, influences the formation of MinDE dynamics and that MinDE pole-to-pole oscillations are a result of geometry sensitivity (see below) [80, 82, 85]. MinDE dynamics are not only influenced by the membrane properties, but also they themselves influence the membrane’s physical properties, a common theme for amphipathic helices [86]. The MTS of both MinD and MinE have been demonstrated to deform liposomes [43, 60, 87, 88], stabilize lipid domains [89], change the membrane viscosity in vitro [90] and even to induce waves of labeled lipids [91]. Lipid membranes in vivo do not only have characteristic physical properties, but also bear a (trans)membrane potential. Although it has been suggested that the MinCDE system is modulated by this membrane potential, oscillations can be reconstituted in vitro in the absence of a potential [92, 93].

## Pattern formation by the MinCDE system

**Fig. 2:**
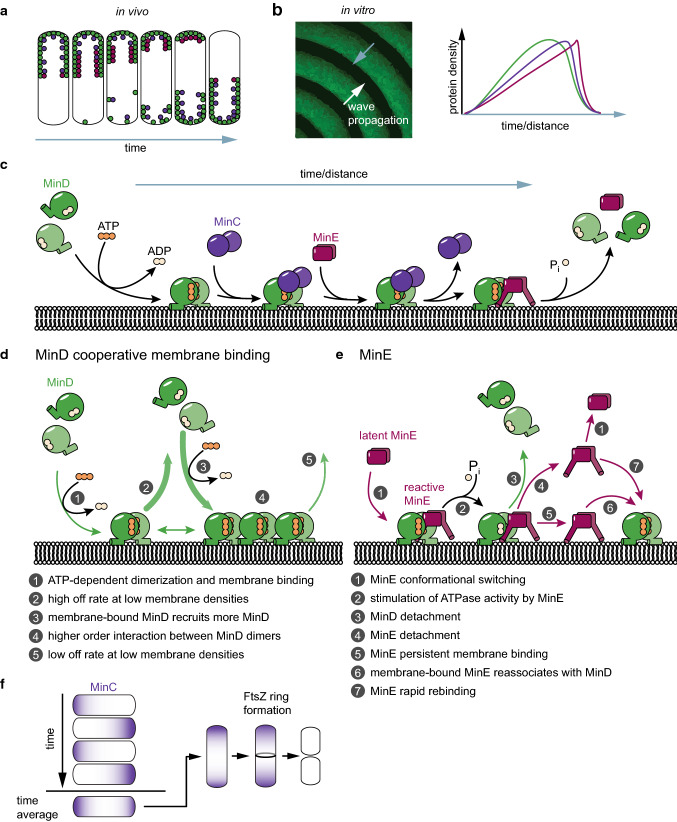
The mechanism of MinCDE pattern formation in vivo and in vitro. **a** Schematic MinCDE oscillation cycle in vivo. **b** Schematic representation of MinCDE dynamics in vitro. Gray arrows indicate the direction of the mechanism displayed in **e**. **c**–**e** Illustration of the mechanistic details of MinDE pattern formation. **f** MinCDE oscillation cycles in vivo result in a time-averaged protein gradient of MinC, that is minimal at midcell and maximal at the compartment poles. For a detailed description, see the main text

The three proteins MinCDE and the membrane act together to form an oscillation cycle. In vivo, the proteins perform pole-to-pole oscillations [18, 19], whereas on a planar membrane in vitro, they form traveling surface waves and other patterns (Fig. 2a, b) [20, 48, 63, 94]. These dynamics, even though of different appearances, share the same basic mechanism (Fig. 2c). Note that MinC is not required for pattern formation, but is only a passive passenger of the MinDE dynamics. The first part of the mechanism is dominated by MinD cooperative membrane binding (Fig. 2d). MinD dimerizes in an ATP-dependent fashion, enabling it to bind to the membrane via its MTS [43, 44, 52]. At the beginning of a MinDE wave or a MinDE oscillation cycle, MinD density is low, and individual MinD dimers rapidly diffuse on the membrane with short residence times [45, 48]. However, MinD membrane binding is highly cooperative, rapidly leading to an increase in MinD density on the membrane towards the end of a MinDE wave or a MinDE oscillation cycle [20, 44, 46, 48]. In this high density region, lateral diffusion of MinD dimers is reduced and membrane residence time is increased [45, 48]. This behavior likely originates from the association of MinD dimers into higher-order structures [42, 43, 45, 47]. MinC dimers associate with membrane-bound MinD in a presumably 1:1 interaction, closely emulating MinD density [19, 48, 78]. MinC is far less abundant than MinDE in the cell; so for every MinC dimer, there would in principle be 10–30 MinD dimers available for binding (see Table 1). However, MinC might not be homogenously distributed when attached to membrane-bound MinD (see discussion on MinCD copolymers in section Positioning of FtsZ).

**Table 1:** Protein concentrations of MinCDE and FtsZ in vivo

	Proteins/cell proteomics	Proteins/cell Western blotting	Concentration [µM] (assuming *E*. *coli* volume of 4.3 fl [142])
MinD	4928 [142]	2000–3000 [49, 227]	0.8–1.9
MinE	4004 [142]	1400 [227]	0.5–1.5
MinC	163 [142]	400 [228]	0.06–0.15
FtsZ	7898 [142]	3200 [143], 5000 [144], 15,000 [145]	1.2–5.6

The second part of the mechanism is dominated by MinE (Fig. 2e). The homodimeric MinE diffuses rapidly in solution when in the closed/latent conformation [51, 52, 59, 66, 67]. Once this latent MinE senses a membrane-bound MinD, it switches into an open, reactive conformation where both the MTS as well as the MinD contact helix are exposed [66, 67]. This open/reactive conformation is able to bind to membrane-bound MinD via the contact helix forming an asymmetric complex. If the MinD dimer is bound by MinC, MinE displaces MinC into solution [44, 77]. In the MinDE complex, MinE stimulates ATPase activity of MinD, triggering ATP hydrolysis and monomerization [44, 50, 68], thereby weakening the membrane attachment of MinD. Once the MinD monomers have detached, MinE has several possible modes of action. (1) MinE could stay on the membrane, termed persistent membrane binding, where it could re-associate with other membrane-bound MinD dimers [48, 52, 67]. (2) MinE also detaches, but before it switches back into the latent conformation it rapidly binds to other membrane-bound MinD dimers, termed rapid rebinding [48]. (3) MinE detaches and switches back into the latent conformation, which can rapidly diffuse away [66, 67]. These three modes of action ensure the local accumulation of MinE in high-MinD-density regions, which will trigger the cooperative MinD detachment. It is important to note that the individual molecules do not move directionally on the membrane, but simply attach and detach in a coordinated fashion governed by the molecular interactions described above [20, 48].

As mentioned, MinC itself is not needed for the generation of patterns. MinDE oscillate in vivo and in vitro without MinC, and the presence of physiological amounts of MinC has a negligible effect on MinDE oscillations [20, 48, 54, 74, 78]. However, in vivo, functional MinC is required to prevent the minicell phenotype [15, 18]. For correct positioning of the division site to midcell, the MinC oscillation powered by MinDE generates a time-averaged protein gradient of MinC (as well as MinD) with maximum concentration at the poles and minimal concentration at the cell center [21, 95]. This gradient acts as a cue for the patterning of downstream targets (see section The MinCDE system in the generation of downstream patterns). With MinC being an inhibitor of FtsZ, the pole-to-pole oscillations confine the inhibitory activity of MinC to the cell poles, restricting FtsZ assembly to midcell (Fig. 2f). However, also a strong MinD gradient with maximum MinD density at the poles could potentially position membrane proteins to midcell via a non-specific mechanism.

### MinCDE oscillations in vivo

**Fig. 3:**
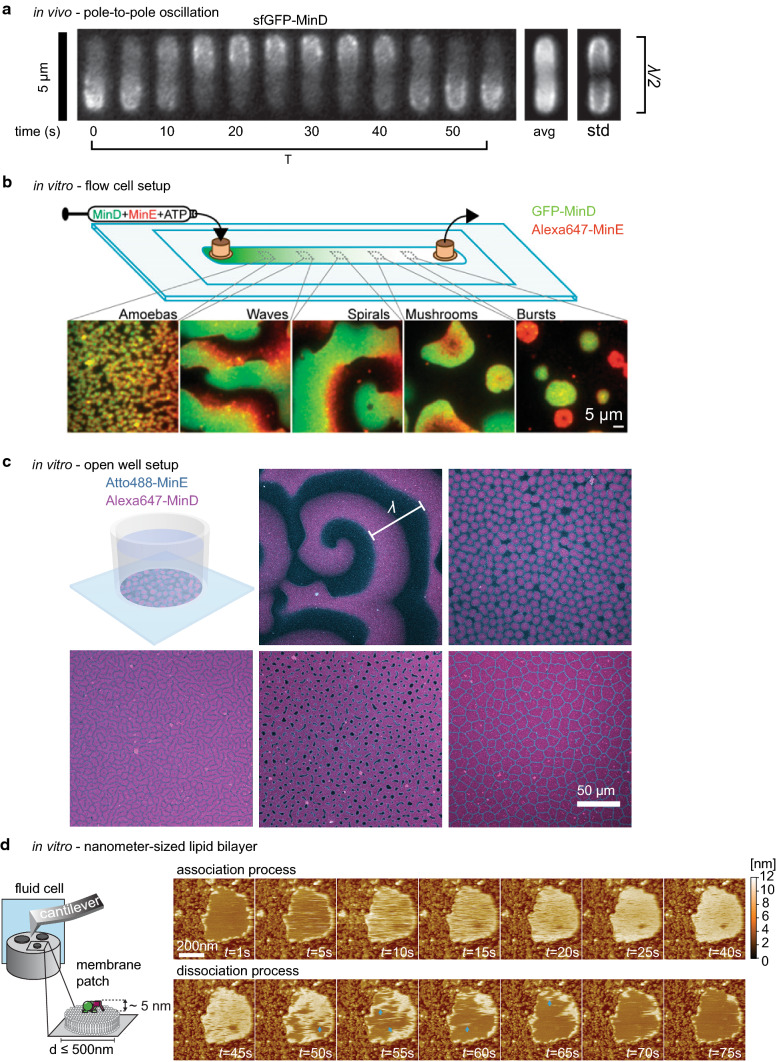
Pattern formation by the MinCDE system. **a** MinCDE perform pole-to-pole oscillations in vivo that lead to a time-averaged protein gradient of MinC and MinD (adapted from [95] by permission from Springer Nature Customer Service Centre GmbH: Springer Nature, Nature Nanotechnology [95], Copyright (2015)). **b** Patterns formed by MinDE in a flow-cell setup in vitro (adapted from [63]). **c** MinDE form traveling surface waves and a variety of stationary patterns in an open well setup in vitro (adapted with permission from [94]. Copyright (2019) American Chemical Society.) **d** MinDE point oscillations on nanometer-sized membranes in vitro as observed with high-speed atomic force microscopy. Blue arrows indicate higher-order structures. (adapted with permission from [45]. Copyright (2018) American Chemical Society.)

Through the mechanism described above, MinD and thereby MinC oscillate from pole to pole within *E*. *coli* (Figs. 2a, 3a) [18, 19, 78]. MinE drives this oscillation, forming a high-density zone at the rim of the retreating MinCD polar zones [71, 96]. This high-density MinE region is also referred to as the MinE ring, and was originally thought to be a static and polymerized structure [70]. MinCDE pole-to-pole oscillations are crucial for the correct positioning of the division site to midcell. Hence, in the complete absence of MinCDE, the typical minicell phenotype occurs, where cells frequently divide asymmetrically, giving rise to anucleate minicells [15]. The same is true for deletion of either MinC or MinD [15]. In contrast, somewhat counterintuitive, in the absence of MinE or with MinE mutants defective for MinD interaction, cells become filamentous. No oscillations occur and MinCD bind to the membrane over the entire length, blocking cell division also at midcell [18, 61]. The same effect can be produced in cells harboring MinD D40A as a sole copy, a MinD mutant deficient for ATP hydrolysis, or MinD D152A, a mutant whose ATPase activity cannot be stimulated by MinE [36, 38]. These results demonstrate that the MinDE pattern formation is crucial to induce a MinC gradient capable of regulating cell division.

In general, oscillations can be characterized by the oscillation period T, the wavelength λ and the wave velocity v (Table 2). The MinDE oscillation period has been extensively characterized in different strains and ranges between 40 and 120 s [18, 71, 78, 95, 96]. In a strain with a functional sfGFP–MinD fusion at the native locus, it was measured to be 68 s at 27 °C [95]. The period of the MinDE oscillation depends on several factors, such as concentration ratios or temperature (Table 2). For instance, a temperature increase was shown to accelerate the molecular dynamics, hence decreasing the oscillation period [97]. Conversely, high MinD/MinE ratios slow dynamics, thus increasing the oscillation period and vice versa [18]. Also the level of MinE-stimulated ATPase activity influences the oscillation frequency, where lower levels of stimulation increase the period [50]. However, a MinE version, found to be hyperactive in MinD ATPase stimulation, displayed significantly slower MinDE oscillations in vivo [94]. This suggests that also other properties of MinE influence the oscillation period [94].

**Table 2:** Parameters defining MinCDE oscillations in vivo and in vitro

	In vivo	Influences on parameter in vivo	In vitro on SLBs	Influences on parameters in vitro
Wavelength (µm)	8–11 µm [18, 97]		30–100 µm [20, 42, 48, 64, 80, 82, 100, 102]	↓ with crowding agents [100, 101, 103], ↑ with membrane fluidity [102], ↓ with increasing MinE concentration [20, 64, 82], ↓with limited protein amount [82, 100], ↓with increasing anionic lipid density [80, 82], ↑with increasing salt concentration [82]
Oscillation period T (s)	40–120 s [18, 71, 78, 95, 96]; 68 s @27 °C [95]	↑with high DE ratios ↑ with lower ATPase stimulation by MinE [50] ↓increasing temperature [97]	40–120 s [20, 42, 48, 64, 80, 100, 102]	
Wave velocity (µm/s)			0.1–0.6 [20, 42, 48, 64, 80, 82, 100, 102]	↑ with increasing MinE concentration [20, 64, 82], ↑with increasing temperature [100] ↓with limited protein amount [82, 100] ↓with increasing anionic lipid density [80, 82] ↑with increasing salt concentration [82]

In contrast to the oscillation period, the wavelength has rarely been determined in vivo. The wavelength of an oscillation is defined as the distance between two similar points. As such, the distance between the maxima of a pole to pole oscillation equals half a wavelength and the distance between two peaks in filamentous long cells equals one wavelength. Inferred from MinD maxima in filamentous cells, the wavelength has been cited to be between 8 and 11 µm and seems to be unmodified by temperature [18, 97].

Interestingly, MinDE pole-to-pole oscillations are not specific to *E*. *coli*. When MinDE from *E*. *coli* are expressed in *Bacillus subtilis*, they also perform pole-to-pole oscillations, suggesting that MinDE pattern formation is independent of other proteinaceous factors [98]. Furthermore, also other bacterial species harbor MinCDE homologues that oscillate (see below).

### In vitro reconstitution of the MinCDE system

For a long time, the unfavorable size of bacteria for optical microscopy and the severe cell division defects upon MinCDE deletion or manipulation restricted the investigation of the MinDE pattern formation mechanism in vivo. Hence, in vitro reconstitution of MinDE oscillations in 2008 represented a major breakthrough for the understanding of MinDE self-organization [20]. Purified MinE and MinD, in the presence of ATP, were shown to form traveling surface waves on a supported lipid bilayer. These experiments demonstrated that MinD, MinE and a lipid membrane are necessary and sufficient for pattern formation [20]. Similar to the in vivo situation, in the absence of MinE, or when MinD was bound to the non-hydrolyzable ATP analog ATPγS, MinD homogenously covered the membrane and no patterns were formed [20, 48]. Likewise, increasing MinE concentrations led to accelerated dynamics, thus reducing the MinDE wavelength and increasing wave velocity [20, 64, 82]. Similar to the MinE ring in vivo, MinE accumulates at the rear of the MinDE wave, driving the dynamics (Fig. 2b) [20, 48, 82]. Since its establishment, the in vitro reconstitution assay in either its original form with an open well geometry [20, 99] or in a flow-cell setup [63, 82], has elucidated various details about the molecular mechanism of the MinDE oscillations in a controlled fashion: the influence of salt concentration [82], membrane composition [80, 82], flow [82], temperature [100], crowding [100–102], the presence of MinC [48, 80], the role of MinE membrane binding [63, 64] and conformational switching [25]. It further served to demonstrate the spatiotemporal regulation of FtsZ [80, 103] and other membrane components [42, 91] by MinDE waves.

Besides traveling surface waves, a variety of different dynamic patterns could be observed in the flow-chamber setup (Fig. 3b) [63]. The observed patterns had different dynamic appearances and were associated with distinct membrane protein densities. From higher protein densities at the inlet of the flow cell to lower densities at the outlet, patterns appeared that were categorized as amoebas, traveling waves, more regular spiral waves, mushrooms and bursts. Despite the different appearances, these patterns all share the same basic characteristics with traveling surface waves: MinE density peaks in time and space after the highest MinD density [63].

A recent study using the open well setup and a more native MinE protein variant could further observe a variety of stationary patterns that closely resemble “Turing patterns”: spots, mesh, inverse spots, labyrinths and intermediate patterns thereof (Fig. 3c) [94]. These patterns are quasi-stationary, because once established, they usually only undergo subtle and slow changes. However, as demonstrated by FRAP, the MinDE proteins are constantly exchanging within the pattern [94]. To our knowledge, these are the first examples of “Turing patterns” occurring in a reconstituted protein system. Which type of pattern emerged was dependent on protein concentration, but is likely influenced by other parameters such as ionic strength or the nature of the support. Intriguingly, also in these stationary patterns the spatial separation between maximum MinD and maximum MinE density can be observed. Furthermore, the assay demonstrated multistability, a fascinating aspect of the MinDE system also observed in vivo (see below) [104]. Even though the proteins were well mixed at the start, two different kinds of patterns were often observed in the chambers that were either spatially or temporally separated.

The time period of the dynamics in vitro is similar to the oscillations in vivo and ranges between 40 and 120 s [20, 48, 64, 100, 102]. In contrast, the in vitro wavelength of the travelling surface waves was shown to be about 30–100 µm [20, 48, 64, 82, 100, 102], and hence is about 10 times larger than in vivo. Similar to the in vivo oscillations, elevated temperatures accelerate MinDE dynamics, leading to increased wave velocities and therefore shorter periods, but do only have a minor impact on the wavelength [100]. Increasing amounts of anionic lipids in the membrane such as DOPG or ardiolipin decrease wave velocity and wavelength [80, 82]. In contrast, higher salt concentrations increase the wavelength and wave velocity [82]. Limited protein amounts, such as in a flow-cell setup or in geometric confinement, seem to generally slow down the dynamics and decrease the wavelength [82, 100]. The difference between the wavelength in vivo and in vitro is likely due to different solution and membrane diffusion of MinD and MinE in cells and buffer. Indeed, increasing concentration of crowding agents in solution, or proteins crowding the membrane also decreased the wavelength in vitro [42, 100, 101, 103]. In contrast, reconstitution on free-standing bilayers with higher membrane fluidity further increased the wavelength of the MinDE traveling waves [102]. That in vitro MinDE dynamics are also occurring on a much smaller length-scale was recently demonstrated by forming supported lipid bilayer patches of only 50–500 nm in diameter [45]. On these patches, the lateral diffusion is confined and the absolute amount of attaching proteins limited to the surface area. Nevertheless, quasi point oscillations of MinDE occur on such a membrane that were imaged with high-speed atomic force microscopy (Fig. 3d) [45].

## The MinCDE system in interplay with geometric boundaries

Over the years, several models have been proposed to explain why MinCDE robustly oscillate from pole to pole within the elongated *E*. *coli*. Possible explanations have been specific cues at the poles, such as specific lipid composition [46] or high membrane curvature [105], or simply the choice of the longest possible distance in the cell as oscillation axis [106]. Various in vivo and in vitro experiments have recently demonstrated that geometry sensing is an intrinsic property of the MinCDE system and originates from the self-organization mechanism itself.

### The MinCDE system as a ruler of cell size

In short *E*. *coli* cells with a length smaller than 2.5–2.7 µm, MinCDE exhibit stochastic fluctuations switching irregularly between cell poles [95, 107]. In contrast, MinCDE reliably oscillate from pole to pole in longer *E*. *coli* cells. Modeling suggests that ATP consumption of the MinCDE system is significantly lower for stochastic switching than for pole-to-pole oscillations. This indicates that the MinCDE system only starts to oscillate prior to division in cells that have reached a sufficient length to preserve energy in shorter cells [107]. A second switch in the oscillation pattern seems to occur shortly before or at the time of division. MinC and MinD have been shown to frequently pause at the septum, switching to a stable double oscillation directly before septum closure [19, 108, 109]. This behavior reflects the geometry sensitivity of the system and ensures equal partitioning of MinCDE into both daughter cells [109]. The behavior of pausing at the septum could also inhibit the over-initiation of FtsZ close to the established septum, similar to what has been reported for the *B*. *subtilis* MinCD/DivIVa/MinJ system (see below) [110, 111].

**Fig. 4:**
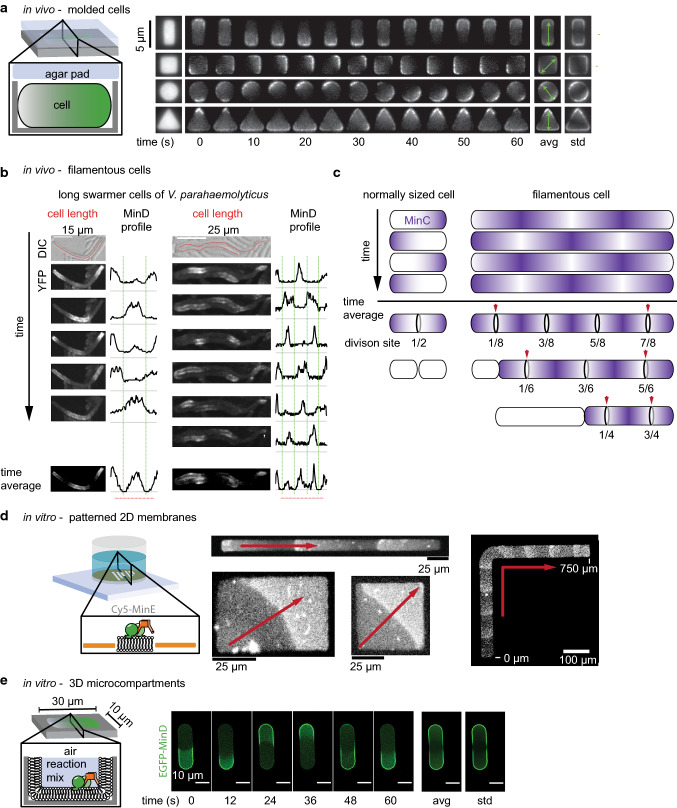
The MinCDE system senses and adapts to geometric boundaries. **a** MinDE oscillations in molded *E*. *coli* cells preferentially orient along a symmetry axis (adapted from [95] by permission from Springer Nature Customer Service Centre GmbH: Springer Nature, Nature Nanotechnology [95], Copyright (2015)). **b** Visualization of MinC oscillations in long *V*. *parahaemolyticus* cells. In the elongated cells, MinCDE forms multi-node standing waves resulting in multiple MinC minima and maxima (adapted from [116] under the Creative Commons Attribution Non-Commercial NoDerivatives License CC BY-NC-ND by permission from John Wiley & Sons Ltd, Copyright (2018)). **c** Schematic representation comparing MinC oscillation and time-averaged gradients in normal-sized and long cells. In *E*. *coli* the division machinery assembles in all MinC minima. Blue arrows indicate positions where the single FtsZ ring in *V*. *parahaemolyticus* and *S*. *elongatus* are formed. **d** MinDE patterns align to geometric boundaries on 2D gold-patterned supported bilayers (adapted from [101]). **e** MinDE perform pole-to-pole oscillation in rod-shaped microcompartments lined with a supported lipid bilayer, forming the characteristic time-averaged gradient (adapted from [99] with permission from JoVE)

MinCDE oscillations have been shown to occur in a variety of cellular geometries, exhibiting an array of different patterns. In normal-sized *E*. *coli*, the proteins form pole-to-pole oscillations. In longer cells, in turn, MinCDE form multi-node standing waves [18, 78] or traveling waves [72]. In rodA deficient, round *E*. *coli* cells, MinCDE either form traveling waves or oscillations with no preferential axis [106]. In penicillin binding protein deficient, branched *E*. *coli* cells with at least three poles, MinCDE oscillate in a circular motion from one branch to the neighboring [112]. In squeezed, aberrantly shaped *E*. *coli*, MinCDE adapt various patterns [113]. To systematically investigate the geometry sensitivity of the MinCDE system, a recent study molded cells into defined shapes (Fig. 4a) [95]. For this purpose, cells were grown in nanofabricated PDMS chambers and treated with A22 and Cephalexin inhibiting rod-shape maintenance and cell wall constriction, respectively. Under these conditions, the cells grew without division and adapted to the shape of the PDMS chamber. The authors observed MinCDE oscillations in diverse shapes: triangles, spheres, rectangles and squares (Fig. 4a).

Intriguingly, MinCDE oscillations preferentially oriented along the symmetry axis. In squares of increasing size, the authors observed a change in the MinCDE oscillations from a rotational movement to an oscillation along the diagonal axis, to a side-to-side oscillation and a three-node standing wave pattern. In rectangular shapes, MinCDE reliably oscillated along the long axis of the cell unless the width was increased to more than 3.5 µm. In this case, MinCDE displayed a second mode of oscillation along the short axis. These two oscillation modes coexisted in a specific size range (width 5 µm; length: 7–11 µm), demonstrating the multi-stability of the system in vivo [104]. However, once a certain oscillation mode was established in a cell, patterns rarely interconverted, despite perturbations occurring during cell growth, demonstrating the robustness of cellular MinCDE oscillations [104]. In rectangular shapes with an aspect ratio similar to growing *E*. *coli* cells (width: < 3 µm, length: 3–6 µm), oscillations almost exclusively occurred along the long axis and the resulting time-averaged gradient displayed a remarkable accuracy. Only above a length of 7 µm, three-node standing waves emerged. This demonstrates that the intrinsic wavelength of the MinCDE system can be adjusted to a certain degree to generate a gradient that scales with cell length. In contrast, the temporal period was largely invariant with respect to cell size. Using a previously introduced model [24], the authors could further show that the most important parameter for symmetry selection is the MinD self-recruitment rate, a parameter describing the cooperative binding of MinD to the membrane [95]. In the future, it would be interesting to see if MinCDE geometry sensitivity is disturbed in mutants impaired for self-interaction.

Until recently, it was not quite clear why cells employ an oscillating Min system that is also capable of sensing the geometry. This is especially intriguing knowing that also rather static gradients of cell division inhibitors such as ParA-type ATPases or the MinCDJ/DivIVa system in *B*. *subtilis* allow for a precise targeting of the divisome to mid-cell. However, three recent studies demonstrate that the oscillating nature of the MinCDE system allows for the division of longer and filamentous cells, ensuring proper distribution of the genetic content. These studies examined cell division and MinCDE oscillations in elongated cells. The first study analyzed division in filamentous *E*. *coli* cells recovering from stress by elevated temperatures, antibiotic treatment or overexpression of SulA [114]. Similarly, another study analyzed division in filamentous cells of the cyanobacterium *Synechococcus elongatus* recovering from stress of growth under dim light conditions [115]. The third study took advantage of a bacterial species that naturally occurs in different lengths: the gram negative bacterium *Vibrio parahaemolyticus* that can differentiate into short swimmer cells and longer swarmer cells [116]. They analyzed division in the elongated swarmer cells.

All three studies found that the cells switch from symmetric cell division in normal-sized cells, resulting in two equal-sized daughter cells, to asymmetric cell division in elongated cells, typically resulting in normal sized and longer cells. They further revealed that MinCDE oscillations defined the potential division sites in shorter and longer cells alike. Intriguingly, the switch was shown to be induced by a change of MinCDE dynamics from pole-to-pole oscillations in normally sized cells to multi-node standing waves and traveling waves in longer cells (Fig. 4b, c). The resulting MinC minima coincided for short and long cells with potential division sites.

In *E*. *coli*, the division machinery formed at all potential division sites, i.e., in MinC minima, as visualized by FtsA localization [114] (Fig. 4c). Division of these filamentous *E*. *coli* cells also occurred apparently randomly at one of these division sites, yielding two cells of normal size or multiples thereof. When this division event or growth changed the length of the cell, such that one MinC minimum more or less would fit in, FtsA rings rapidly reorganized [114].

In contrast, in both *S*. *elongatus* and *V*. *parahaemolyticus*, the division machinery only assembled in one of the MinC minima, as visualized by FtsZ localization [115, 116]. This single division site was usually found closest to one of the two cell poles, approximately spaced one normal cell length from the pole (red arrows in Fig. 4c). Correspondingly, both *V*. *haemolyticus* and *S*. *elongatus* also divided at one of the pole-proximal division sites, resulting in a normal-sized and an elongated cell. For *V*. *parahaemolyticus*, it could be shown that the limitation to one division site is presumably due to a regulation of FtsZ levels. Short and long swarmer cells had similar FtsZ levels, presumably only allowing one FtsZ ring to form [116]. However, it is unclear why this single FtsZ ring forms and subsequent division occurs preferably at one of the pole-proximal sites. One explanation could be the transient occurrence of traveling waves that have been directly observed in *S*. *elongatus* [115], but have also been previously reported for filamentous *E*. *coli* cells [72]. These traveling MinCDE waves would only preserve one stable MinC minimum at the pole where the waves originate.

These three studies demonstrate that the MinCDE system, in both short and long cells, is an efficient ruler of cell size. This was beautifully visualized by the sudden reorganization of FtsA-marked division sites upon cell length changes due to growth or division [114]. Also in elongated cells, the MinCDE system still lives up to its name, preventing minicell formation: The MinCDE system protects cell poles from cell division and ensures the generation of cells that are either of normal size or multiples thereof to maintain genomic integrity. Not only does the switch in MinCDE oscillations ensure correct cell size distribution, but also allows to maintain a population that contains shorter and longer cells alike. This size plasticity can be beneficial in the face of environmental stress or as a virulence strategy [117].

The inherent geometry sensitivity of the MinCDE system could also explain how MinCDE could have evolutionarily adapted in bacterial cells with other shapes, e.g. in the round cocci *Neisseria gonorrhoeae* [118, 119]. The MinCDE system further presents a likely candidate for FtsZ positioning in the extraordinarily shaped species of the Marine Oligochaete and Nematode Thiotrophic Symbionts (MONTS) cluster of *Gammaproteobacteria*. Some of these gammaproteobacteria are extremely elongated with a length of 45–120 µm [120], whereas others localize FtsZ and divide along the longitudinal cell axis [121].

All in all, the oscillating behavior of the MinCDE system and its geometry sensitivity support an efficient usage of ATP in small cells, the equal partitioning of MinCDE into daughter cells and division of normal-sized and filamentous cells alike. Geometry sensitivity might further allow the system to adapt to different cell geometries.

### Geometry sensitivity of the MinDE system in vitro

The MinDE system’s geometry sensitivity in vitro has been studied in depth. The first demonstration that it is an inherent property of the MinDE system, and thus also occurs in vitro, was the use of planar, but patterned supported lipid bilayers (Fig. 4d) [101]. Intriguingly, MinDE traveling waves were shown to respond to the 2D boundaries by aligning to the shapes. Similar to MinDE waves in cells that were molded into specific shapes [95], MinDE waves in vitro also align to the diagonal on membrane squares or rectangles with high aspect ratio. In contrast, rectangular forms with an aspect ratio below 0.3 induced an alignment to the long axis [101]. On membrane strips shaped like an L or a serpentine MinDE waves could thus be guided by the geometric form (Fig. 4d) [101]. These experiments further indicated that the characteristic in vivo pole-to-pole oscillations arise from an interplay of the MinDE system with the cell’s geometry, but that reproduction of this behavior requires spatial confinement. Indeed, the MinDE system was shown to perform pole-to-pole oscillations in vitro when confined in rod-shaped microcompartments clad with a supported lipid bilayer and with dimensions adjusted to the larger wavelength of MinDE in vitro (10 × 30 µm) (Fig. 4e) [80, 85]. In these compartments also the characteristic time-averaged gradient of MinC and MinD emerged, with highest concentrations at the compartment poles and minimal concentration at the compartment middle [64, 80, 99]. Analogous to the results of MinDE oscillations in molded cells [95], pole-to-pole oscillations in vitro were the prevailing oscillation mode in the rod-shaped compartments with length between 15 and 35 µm. This again demonstrated a certain plasticity of the MinDE wavelength [80]. Only in even longer microcompartments resembling filamentous cells, MinDE formed multi-node standing waves that produced several MinC minima [80, 85]. In round microcompartments mimicking spherical cells, MinDE oscillations had no preferential oscillation axis [80, 85]. While these compartments were closed by an air interface instead of a bilayer, confinement of MinDE in 3D microcompartments covered with a supported lipid bilayer produced similar pole-to-pole oscillations when the aspect ratio and size of the compartment matched those of an *E*. *coli* cell scaled to the in vitro MinDE wavelength [100].

More recent studies have worked towards the reconstitution of MinDE oscillations in fully confined and non-supported 3D geometries [88, 122]. In spherical water-in-oil droplets with a lipid monolayer or giant unilamellar vesicles, MinDE exhibit distinct modes of oscillations: a pulsing mode, pole-to-pole oscillations, circling waves and trigger waves [88, 122]. Deformation of these droplets and vesicles towards more cell-like nonspherical geometry is still a pending experimental goal.

In summary, in vitro the interplay of the MinCDE system with geometric boundaries allows to guide MinDE traveling waves and to mimic the pole-to-pole oscillations occurring in vivo.

## MinCDE system in the generation of downstream patterns

Importantly, MinCDE patterns dictate the spatiotemporal localization of target molecules: (1) MinDE oscillations position MinC and thereby confine divisome formation to midcell; (2) MinDE oscillations non-specifically regulate other membrane proteins in vitro; (3) MinCDE oscillations might participate in chromosome segregation.

### Positioning of FtsZ

The *E*. *coli* divisome is a multi-protein machinery that is coordinated by the tubulin homologue FtsZ [123–125]. Upon GTP binding, FtsZ polymerizes in a head-to-tail fashion [126]. Interestingly, the kinetic polarity of FtsZ filaments has recently been reported to be opposite of microtubules: In FtsZ filaments, incoming subunits seem to preferentially attach to the C-terminal domain of FtsZ (plus end in FtsZ; minus end in microtubule) and not at the GTP-bound N-terminal domain (minus end in FtsZ; plus end in microtubule) [127]. These protofilaments assemble at the future division site in a ring-like structure, recruiting several other components [123, 124]. FtsZ filaments are anchored to the membrane via FtsA and ZipA, a multimeric, peripheral membrane protein with a membrane-targeting sequence, and a single-pass transmembrane protein, respectively [128, 129]. Both anchors interact with the C-terminal peptide of FtsZ [130, 131]. On the membrane, FtsZ filaments treadmill in a GTP-dependent fashion in vivo and in vitro [132, 133]. Recently, the treadmilling activity has been shown to drive the processive, circumferential movement of peptidoglycan synthetases around the septum, thereby ensuring homogenous insertion of new peptidoglycan [133, 134]. FtsZ filaments have further been shown to deform membranes in vitro, which led to the suggestion that the filaments themselves exert the force required for membrane constriction [135]. Regardless whether FtsZ itself provides force for septum constriction or only indirectly assists by guiding the septal cell wall synthetases, it is the key player and as such, the target for spatiotemporal regulation of cell division (for detailed reviews on the divisome see [123, 124]). *E*. *coli* harbors two negative regulators of FtsZ assembly, i.e., inhibitors, the MinCDE system discussed here and the nucleoid occlusion system, where the nucleoid-associated SlmA prevents FtsZ assembly over the chromosome [136].

As discussed above, MinC itself is not needed for the generation of patterns, but is as inhibitor of FtsZ assembly the actual biological agent of the system. The output of the MinCDE pole-to-pole oscillations is a time-averaged gradient of this inhibitor with concentration maxima at the poles and a minimum at the cell center, restricting FtsZ assembly to midcell [21, 95]. But what is the molecular mechanism of MinC antagonizing FtsZ? Overproduction of MinC in the presence or absence of MinCDE inhibits the assembly of FtsZ rings over the entire length of the cell, causing cell filamentation [15, 74, 137]. In vitro, MinC prevents sedimentation of FtsZ filaments by shortening the length of protofilaments [74, 76, 138, 139]. Interestingly, MinC does so without influencing GTPase activity of FtsZ [74, 138, 139], but FtsZ filaments stabilized with a non-hydrolyzable GTP analog, GMPCPP, are not disassembled by MinC [76, 139, 140]. This suggests that while MinC does not affect GTPase activity of FtsZ, FtsZ still needs to be GTPase active and hence, undergo dynamic subunit turnover to be inhibited by MinC.

As MinC consists of two domains that both harbor FtsZ inhibitory activity, researchers have analyzed the individual domains to better understand the action of the full-length, dimeric protein. The N-terminal domain of MinC (MinC-N) alone behaves similar to MinC in vivo: it inhibits cell division, leading to cell filamentation when overexpressed both in the presence or absence of wild-type MinCDE [73]. MinC-N also harbors the activity to prevent FtsZ sedimentation, i.e. shorten FtsZ protofilaments in vitro [73, 138]. Mutational studies revealed that MinC-N interacts with the helix 10 in the C-terminal domain of FtsZ, which is part of the interface between subunits and not solvent-accessible in GTP-bound FtsZ protofilaments [126, 138, 141]. Subsequent studies showed that MinC-N or MinC bind FtsZ–GDP monomers in a one-to-one complex in solution. This suggests that MinC sequesters FtsZ monomers, thereby decreasing the assembly rate, which leads to shorter filaments [139, 141]. However, under physiological conditions within the cell, FtsZ monomers outnumber MinC at least 40-fold and MinC is tethered to the membrane by MinD (see Table 1) [142–145]. Hence, subsequent studies suggested MinC-N to interact with FtsZ subunits where the helix 10 is accessible: with subunits where the C-terminal domain of FtsZ is exposed, i.e., at the plus end of a filament, or with FtsZ–GDP subunits located within the filament, where intersubunit contacts might be weakened [138, 140, 141, 146]. Both mechanisms seem to be plausible: FtsZ turnover regularly exposes filament ends in the FtsZ bundles, and FtsZ protofilaments contain a significant portion of GDP-bound subunits [140, 147]. Such an interaction could either block the attachment of new FtsZ subunits to the plus end of the filament, lead to the accelerated detachment of FtsZ–GDP subunits, and/or break filaments at a binding site [138, 140, 141, 146]. Note that direct proof for a breakage of filaments by MinC is still lacking.

In contrast to MinC-N, the C-terminal domain of MinC (MinC-C) only induces filamentation when overproduced in the presence of MinD or MinDE, but not in the absence of MinD [75]. In vitro, MinC-C cannot prevent FtsZ sedimentation but has been reported to bind to GMPCPP-stabilized filaments and to prevent the lateral association of filaments [76]. Indeed, when expressed at moderate levels in the absence of MinE, MinC-C colocalizes with FtsZ rings and recruits MinD [148, 149]. Furthermore, MinC-C pole-to-pole oscillations in the presence of MinDE pause at the FtsZ ring [148] and MinC mutants unable to inhibit FtsZ assembly were recently shown to exhibit slowed pole-to-pole oscillations [146]. Like FtsA and ZipA, MinC-C interacts with the conserved C-terminal peptide of FtsZ (CCTP) [149] and recruitment of MinC-C and MinD to the FtsZ ring has been shown to displace the FtsZ anchor FtsA from the Z-ring [149].

Notably, the activity of MinC-C in vivo largely depends on MinD. Also, the inhibitory activity of full-length MinC is strongly activated by MinD: MinC acts as a potent inhibitor of FtsZ when bound to MinD, which in the absence of MinE leads to a block in cell division and cell filamentation [15]. However, in the absence of MinD, MinC needs to be overexpressed 25- to 50-fold to achieve a similar cell division defect [137]. Where does this activation come from? One explanation is that through binding of MinD, MinC is removed from the cytoplasm and recruited to the membrane, thereby increasing its effective concentration in close proximity to the membrane-anchored FtsZ filaments [19, 78]. This explanation is supported by in vivo experiments where MinC was directly targeted to the membrane via the MTS of the *B*. *subtilis* MinD or the transmembrane domain of ZipA, which impeded division even in the absence of MinD [41, 150]. However, the inhibitory activity of the membrane-tethered ZipA–MinC fusion could further be increased by coexpression of MinD [150]. This indicates that MinD not only enriches MinC on the membrane, but also activates MinC via a second mechanism.

Recently, it was proposed that the MinD-dependent MinC activation is caused by MinCD copolymer formation [54, 151]. MinCD copolymers are filaments formed in the presence of ATP in vitro, that consist of alternating MinD and MinC dimers [54, 151]. Importantly, these polymers have been shown to form in the absence of membranes in vitro, but the presence of membranes reduced the protein concentrations required for polymerization [54, 151]. In contrast, higher-order structures or filaments formed by MinD have only been observed in the presence of membranes [43, 45, 47]. Addition of MinE to the MinCD copolymers in vitro leads to their rapid disassembly, indicating that the proteins retain functionality [54, 151]. As MinDE oscillations are largely unaltered by MinC under physiological conditions, these copolymers cannot play a role in the oscillatory mechanism itself [19, 48, 54, 78]. Instead, it was suggested that MinCD copolymers bind to FtsZ filaments with higher affinity than to monomers, increasing the inhibitory activity of MinC [54]. Such MinCD copolymers could also exist in vivo as MinCD, in the absence of MinE, are not always homogenously distributed along the membrane [19, 78], but sometimes also display a patchy appearance [54, 148]. However, the importance of MinCD copolymers for the interaction with FtsZ has recently been questioned using MinC/MinD heterodimers formed from wild-type monomers and monomers deficient for copolymer formation [152]. When expressed in conjunction with wildtype MinD/MinC, MinC and MinD were still able to interact via one monomer subunit and efficiently complemented a minicell phenotype [152]. Instead of the necessity for copolymer formation the authors found that the RGSQ motif of MinC and MinD likely form the binding site for the CCTP, either by inducing a conformational change in MinC or directly providing the binding interface [152].

**Fig. 5:**
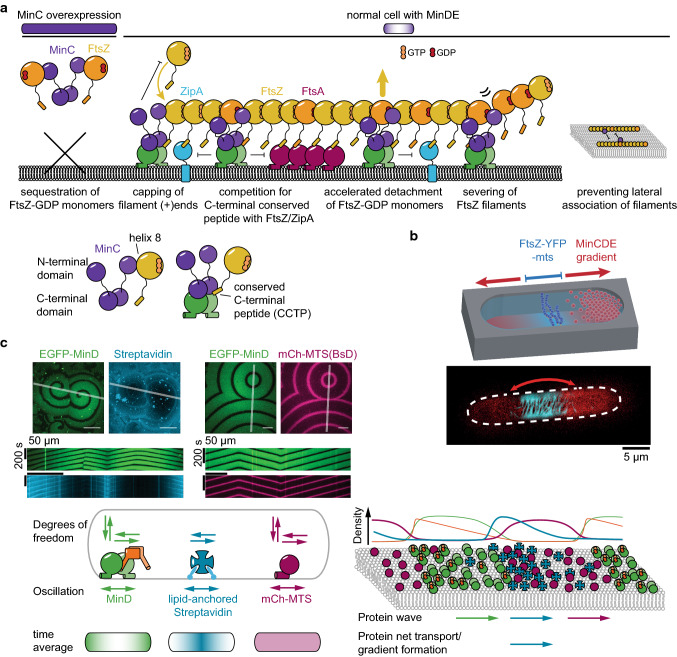
The MinCDE system can induce patterns of other molecules. **a** Scheme explaining the potential modes of action of MinC in filamentous cells, where MinC is heavily overexpressed and in wild-type cells. **b** In vitro reconstitution of MinCDE pole-to-pole oscillations in rod-shaped microcompartments confines FtsZ-YFP-MTS polymerization to the middle zone of the compartment. (adapted from [80] under the CC BY 4.0 license). **c** Spatiotemporal regulation of peripheral (mCh-MTS) or permanently anchored membrane proteins (streptavidin bound to biotinylated lipids) by MinDE in vitro (adapted from [42] under the CC BY 4.0 license)

Also in in vitro reconstitution experiments MinCDE waves are able to spatiotemporally regulate membrane-tethered FtsZ filaments [42, 80, 103, 140]. Intriguingly, when FtsZ is not anchored to the membrane, it follows MinCDE waves [103], demonstrating the capability of MinC to tightly bind FtsZ. MinC-dependent inhibition was also observed when FtsZ-YFP-MTS was used, a chimera that does not harbor the CCTP and can hence bind to the membrane without ZipA/FtsA [42, 80, 140]. In rod-shaped microcompartments, MinCDE oscillations were also able to suppress FtsZ-YFP-MTS assembly at the poles of the compartment (Fig. 5b) [80]. However, MinCDE could only restrict FtsZ-YFP-MTS polymerization to a broad zone in the compartment center. In contrast, in vivo, FtsZ forms a sharp ring with small width, indicating that the in vitro system lacks important factors. Of course, the system is simplified and omits several layers of complexity: (1) the used compartment was open at the top; (2) so far, only the FtsZ-YFP-MTS chimeric protein was used, lacking the CCTP shown to be a target of full-length MinC; (3) a nucleoid mimicry that would allow to establish nucleoid occlusion and crowding is lacking; (4) accessory divisome protein such as the bundling proteins ZapAB are missing; (5) due to the use of FtsZ-YFP-MTS, the native anchor proteins FtsA and ZipA are not present; (6) crowding in solution and on the membrane is absent. The absence of the FtsZ anchors and other membrane proteins in the assay could have the biggest impact, as the potential enhancement of FtsZ ring formation by non-specific regulation through MinDE oscillation, as discussed in the next section, cannot occur.

On the basis of the results discussed above, a model for MinC inhibitory activity emerges. Full-length MinC in solution can sequester FtsZ monomers, a scenario presumably occurring when MinC is heavily overexpressed causing cell filamentation [139, 141]. However, in wild-type cells, MinC levels are low compared to FtsZ, rendering a sequestration-based mechanism unlikely (see Table 1). In this case, full-length MinC will be confined to the membrane by the interaction of MinC-C with MinD that could involve MinCD copolymers [38, 54]. This interaction does not only increase the effective concentration of MinC, but also presumably forms the binding site of MinC-C for the CCTP [152]. MinC-C binding to the CCTP will displace ZipA and FtsA that anchor FtsZ filaments by also interacting with the CCTP [149] and decrease the bundling of filaments presumably through steric hindrance [76]. The N-terminal part of MinC in turn binds to the FtsZ subunits at the plus end of the filaments or to FtsZ–GDP subunits within the filament, where it might either break the polymer, increase FtsZ–GDP detachment, and/or block binding sites for incoming monomers [138, 140, 141, 146].

### Non-specific patterning by MinDE

There have always been speculations whether MinCDE oscillations in *E*. *coli* are involved in other tasks than generating a MinC gradient inhibiting FtsZ assembly. Several in vivo studies point to a role in the positioning and regulation of inner membrane proteins. For example, the polar localization of three foci-forming inner membrane proteins TnaA, GroES and YqjD was disrupted in the absence of MinCDE [153]. Furthermore, the FtsZ anchor ZipA was reported to counter-oscillate to MinCDE, and this did not occur when MinDE were deleted [154]. These counter-oscillations were explained by recruitment of ZipA to FtsZ filaments that are periodically depolymerized by MinDE-driven MinC oscillations, but could as well be explained by a direct positioning of ZipA by MinDE oscillations. A recent study supports this hypothesis: when TIRF microscopy was used to investigate FtsZ treadmilling dynamics, the overall FtsZ dynamics was shown to change when MinCDE were deleted, but not when only MinC was deleted [133]. This suggests that MinDE oscillations change FtsZ dynamics independent of MinC, possibly through regulation of other divisome components such as the FtsZ anchors ZipA and FtsA.

The first direct proof for the regulation of inner membrane proteins by MinCDE oscillations originated from a quantitative proteomics study comparing the abundance of peripheral membrane proteins of a *ΔminCDE* with a wildtype *E*. *coli* strain [155]. This study identified several proteins that had a decreased abundance in the absence of the MinCDE system and demonstrated that they directly interacted with MinCDE. This strongly suggests a direct recruitment of these proteins to the membrane by MinCDE. For proteins that were enriched on the membrane in the absence of the MinCDE system, the authors proposed that MinCDE exclude these proteins from binding to the inner membrane. They could further demonstrate that the abundance of several metabolites differed between the two strains, and that these metabolites were produced by pathways involving the peripheral membrane proteins regulated by MinDE [155].

Taking advantage of the well-established MinCDE in vitro reconstitution assay, two independent studies recently demonstrated that the MinCDE system is indeed capable of regulating and transporting membrane proteins in vitro [42, 91]. Independent of MinC, peripheral membrane proteins were shown to be spatiotemporally regulated by MinDE waves both on planar SLBs as well as in rod-shaped microcompartments. Intriguingly, these proteins performed counter-oscillations to the MinDE oscillations [42]. MinDE waves did not only dictate the localization of the peripheral membrane proteins, but also decreased their overall density on the membrane [42]. These experiments support the previously suggested mechanism by Lee et al.: MinDE displace other peripheral membrane proteins from the membrane [155]. A very similar competition of peripheral membrane proteins, leading to the accumulation of the strongest peripheral membrane proteins and the displacement of weaker peripheral membrane proteins, has been observed in lipid droplet protein compositions in eukaryotes [156]. Interestingly, MinDE waves were also able to displace model peripheral membrane proteins equipped with two copies of the MinD MTS itself, suggesting that MinD indeed assembles into higher-order structures, rather than dimers, on the membrane [42].

Even more intriguingly, both studies showed that MinDE waves can also spatiotemporally regulate permanently attached membrane proteins, in this case lipid-anchored streptavidin [42, 91]. MinDE dynamics established large-scale streptavidin gradients on planar membranes, demonstrating that MinDE waves drive the directed transport of membrane-attached proteins. Similarly, in rod-shaped microcompartments, MinDE pole-to-pole oscillations drove counter-oscillations of lipid-anchored streptavidin. In contrast to the regulation of peripheral membrane proteins, this regulation established a time-averaged protein gradient of lipid-anchored streptavidin. As the gradient was maximal at the center of the compartments and minimal at the poles, the protein is enriched at the compartment center over time [42]. Both studies indicate a unique molecular mechanism underlying this non-specific membrane transport [42, 91]: MinDE waves on the membrane are of such high density, and/or contain higher-order structures, that they represent a steric obstacle to other diffusing membrane proteins. Because the MinDE protein density translocates in a specific direction on the membrane, it constitutes a propagating diffusion barrier that biases the diffusion of other membrane proteins in the direction of wave propagation. MinDE waves were also shown to shift labeled lipids albeit very weakly and without inducing large-scale gradients [91], which is in agreement with earlier reports of MinDE altering the physical properties of membranes [43, 60, 87–90]. This suggests that the MinDE oscillations might also affect membrane protein diffusion and binding by locally modifying the membrane properties, although the effect on lipids may be emphasized when they carry a fluorescent dye. Thus, similar to the actin cortex in eukaryotic cells [157], the circumferentially rotating actin homologue MreB in bacteria [158, 159], or the StpABCD diffusion barrier in the *Caulobacter crescentus* stalk [160], MinDE oscillations locally modify the attachment and diffusion of membrane proteins, albeit in a dynamic fashion.

While the direct visualization of such a MinDE-dependent positioning of membrane proteins is still lacking in vivo, this mechanism would have important implications for the spatiotemporal organization of the cell. MinDE oscillations would lead to the time-averaged accumulation of strongly anchored proteins at the division site, e.g., ZipA and FtsA, thus enhancing cell division. They would further increase the turnover of monomeric peripheral membrane proteins, leading to the mixing of membrane content prior to cell division.

Furthermore, this finding also has implications for other reaction–diffusion systems and intracellular dynamics. A plethora of intracellular waves in eukaryotic cells have been discovered, but their biological role and how these waves confer information remain unclear [161]. These waves originate from positive feedback loops whereby membrane-bound factors such as GTPases or curvature-recognizing proteins and cortical actin rhythmically accumulate. The possibility of MinDE-dependent membrane protein transport suggests that both, the accumulated membrane proteins as well as the cortical actin, could act as propagating diffusion barriers, regulating and transporting other membrane proteins and thereby mixing the cell membrane content.

### A possible link between chromosome segregation and the MinCDE system

Besides the positioning of target molecules by MinDE oscillations, the oscillations might be involved in the positioning and segregation of the entire nucleoid. To date, it is unclear how exactly *E*. *coli* segregates its chromosome (for a comprehensive review of the topic see [162]). *E*. *coli* contains the SMC protein MukB, which together with MukEF, Topoisomerase IV and the Ter macrodomain organizing protein MatP, arranges and decatenates the chromosome [163, 164]. However, these proteins are unlikely the sole players in chromosome segregation. Contrary to other bacterial species, *E*. *coli* lacks any ParABS systems aiding chromosome segregation [165]. Thus, several other models for chromosome segregation have been proposed: based on entropic repulsion [166], through co-transcriptional translation and protein translocation (transertion) [167] or through direct binding of MinD to DNA [168]. MinD is the closest homologue to ParA in *E*. *coli*, and several studies report chromosome segregation defects in MinCDE deletions that cannot be explained by mere cell division defects [168–173]. Most of these reports compare *ΔminB* (deletion of *minCDE*) to wild-type strains, without being able to discern which of the three proteins are important for effective chromosome segregation. However, two studies indeed indicate that intact MinDE oscillations are important for chromosome segregation, as cells showed impaired segregation when either MinE was overexpressed [170] or when all three proteins were deleted, but not when only MinC is deleted [168]. In addition, *E*. *coli* strains deleted for the two subunits hupA and hupB of the nucleoid-associated protein HU acquire secondary mutations in the *minCDE* genes, suggesting a link between chromosome organization and the MinCDE system [174]. Interestingly, longitudinal density waves in hupA-stained *E*. *coli* nucleoids have been observed that occur on the time-scale of MinDE oscillations [175].

All these observations led to the suggestion that MinDE oscillations drive chromosome segregation by direct DNA binding of MinD [168]. A computer simulation considered entropic repulsion of the chromosomes too weak for efficient segregation, but showed that a gradient of DNA–membrane tethering sites along the longitudinal axis of the cell would bias the motion of the chromosome towards the cell poles [168]. The study subsequently showed a weak, non-specific DNA binding of MinD [168]. However, using the in vitro reconstitution of MinDE, no recruitment of DNA to the MinDE waves could be observed [42]. In contrast, DNA targeted to the membrane via a cholesterol anchor or via streptavidin biotin anchors, was spatiotemporally regulated by MinDE, but accumulated in the minima of the MinDE wave and did not co-localize with MinD [42]. These experiments cannot rule out that MinD binds to DNA in vivo, as binding of MinD to DNA might be enhanced or there might exist adaptor proteins linking MinD and the nucleoid in vivo. However, these experiments suggest a twist to the proposed mechanism: Not MinD itself forms the gradient of DNA–membrane tethers, but rather MinDE oscillations could regulate DNA–membrane tethers, thereby establishing oscillating gradients that aid chromosome segregation. Such DNA–membrane tethers are manifold in *E*. *coli*, e.g. membrane-bound transcription factors [176] or nucleoid-membrane contacts occurring during transertion [177]. The latter had been suggested to be involved in chromosome segregation [167]. Interestingly, a recent study showed that in *B*. *subtilis*, MinD seems to be involved in chromosome segregation during sporulation by directly interacting with Soj the ParA ATPase of the chromosome segregation system ParABS [178]. In combination, all these studies provide compelling evidence for a link between MinDE oscillations and chromosome segregation in *E*. *coli*, but the exact mechanism warrants further investigation.

## Differences and similarities to other ParA-type ATPases

### The Min system in other bacterial species

Homologues of all three *E*. *coli* Min proteins, MinCDE, are conserved in several Gram-negative bacteria, suggesting that these species also harbor oscillating protein systems [179]. Indirect proof for the presence of a functional and presumably oscillating MinCDE system arises from the recent surge in the production of minicells for applications (see below), e.g. in *Salmonella typhimurium*, *Shigella flexneri* and *Pseudomonas aeruginosa*, mutation or deletion of the MinCDE locus cause minicell formation [180, 181]. Another species potentially containing an oscillating MinCDE system is the pathogenic coccus *Neisseria gonorrhoeae*, which requires MinD and MinC for normal division and cell morphology [119, 182]. Intriguingly, when expressed in *E*. *coli*, MinD and MinE from *N*. *gonorrhoeae* are able to oscillate within the cell [118]. Recently, MinCDE oscillations have been shown to occur in a number of different bacterial species by directly visualizing them inside those species. For example, in the plant pathogen *Xanthomonas citri*, MinC displayed the characteristic pole-to-pole oscillations and deletion of MinC led to the classical minicell phenotype, defects in chromosome segregation, and also to branching of cells under certain media conditions [183]. Also in the rod-shaped cyanobacterium *S*. *elongatus*, MinCDE oscillate and position FtsZ [115, 184], even though these cells contain complex intracellular thylakoid membranes. The study showed that for robust oscillations to occur, a certain permeability of the intracellular membranes and presumably a selective binding of MinDE to the plasma membrane are required [184]. These results indicate that MinCDE oscillations can serve as a robust spatial cue even in more complex cellular environments [184]. Interestingly, the division machinery of chloroplasts is similar to those of bacteria, harboring homologues of FtsZ, MinD, MinE and a MinC-like inhibitor of FtsZ, ARC3, but it is unknown if MinDE oscillate [185].

Also in the Gram-negative bacterium, *Vibrio parahaemolyticus* that can differentiate in long swarmer and shorter swimmer cells, MinCDE oscillate, thereby preventing polar FtsZ localization and thus, a minicell phenotype [116]. In contrast to all these species, in the multi-chromosomal *Vibrio cholerae*, MinCD were shown to oscillate from pole to pole without influencing the localization of FtsZ, which was even frequently found at one cell pole. Indeed, deletion of MinCDE in *V*. *cholerae* did not result in a strong minicell phenotype or alter FtsZ localization [186]. While *V*. *cholerae* has retained an oscillating MinCDE system, the proteins are not participating in divisome localization in this species, suggesting an alternate role of these oscillations [186].

In most Gram-positive bacteria, only MinC and MinD are conserved and the topological determinant is not MinE, but DivIVa and MinJ that recruit MinCD to the cell poles [187–190]. DivIVA seems to bind to negatively curved membranes, i.e., the cell poles and nascent septa [191–193]. The protein is a coiled coil protein that resembles the curved form of eukaryotic BAR domains and might sense membrane curvature in a similar fashion [194]. The integral membrane protein MinJ is recruited to DivIVa, serving as an adapter to further recruit MinD and thereby MinC [189, 190]. Accordingly, the *B*. *subtilis* Min system, like the *E*. *coli* Min system, was believed to mainly inhibit FtsZ assembly at the cell poles, just that MinCD do not oscillate. Instead, they appear to be statically localized at the cell poles and septa via DivIVA and MinJ, forming a protein gradient that is minimal at the cell centre [187]. However, the concept of a static MinCDJ/DivIVa system has been changing. DivIVa primarily localizes at newly formed division sites and the observed enrichments at the poles seem to be remnants from former septa [111, 195]. Indeed, DivIVA as well as MinC have been shown to dynamically relocate from the poles to newly formed septa, with MinC arriving later and leaving earlier [110, 111]. *The B*. *subtilis* MinD has been shown to bind to the membrane via its conserved C-terminal MTS, exhibiting dynamics that are consistent with diffusion on the membrane and/or dissociation and association processes [39, 196].

These observations suggest that in contrast to the *E*. *coli* MinCDE system, MinCDJ/DivIVa in *B*. *subtilis* do not prevent FtsZ assembly at old poles, but rather prevent over-initiation, i.e., the formation of multiple neighboring septa at the division site. Besides, the origin of the slow protein dynamics and the role of the ATPase MinD in this system remains elusive. If MinD were only to be a passive adapter between MinC and DivIVa/MinJ, then why does the protein contain an amphipathic helix allowing it to bind to the membrane by itself [39, 196] and why is its ATPase activity crucial for the MinCDJ/DivIVa system [197]? Interestingly, expression of MinD and MinE from *E*. *coli* in *B*. *subtilis* in the absence of either MinC inhibited spore formation, presumably through inhibition of polar septum formation [98]. This suggests that an oscillating Min system is incompatible with spore formation, which could be either because MinDE oscillations abrogate the interaction of MinD and Soj involved in chromosome segregation during sporulation [178], or MinDE oscillations spatiotemporally regulate other membrane-bound divisome components as described above. However, a close relative of *B*. *subtilis*, the Gram-positive spore-forming bacterium *Clostridium difficile*, contains all three proteins MinCDE and DivIVa, but no MinJ [198]. When expressed in *B*. *subtilis*, *C*. *difficile* MinD and MinE also performed pole-to-pole oscillations and inhibited spore formation [199]. Furthermore, DivIVa and MinD from *C*. *difficile* have been shown to directly interact, indicating that an oscillating MinDE system and the presence of DivIVA do not exclude each other [198]. Although the exact mechanism of the MinCDJ/DivIVA system remains to be elucidated, it might be based on an interplay of curvature recognition and a reaction–diffusion-based mechanism.

Despite our rather detailed knowledge about prokaryotic cell division, so far not much is known about how archaea, representing the third domain of life, control and perform cell division (for a detailed review see [200]). Depending on the phylum, archaea harbor different homologues to either eukaryotic (ESCRT-III) [201] or prokaryotic proteins (FtsZ) [202, 203] that have been implicated in cell division. For example in euryarchaeota, FtsZ homologues localize to the future division site [204, 205]. Archaea belonging to this phylum also encode homologues to the known regulators of FtsZ in prokaryotes, MinD and ParA-type ATPases, indicating that division site selection could be similar. Indeed the euryarchaeal rod-shaped archaeon *Halobacterium salinarum* divides in a similar fashion to *E*. *coli*, according to the adder principle [206]. Even more intriguingly the pleomorphic archaea *Haloferax volcanii* and *Haloarcula japonica*, that assume complex shapes such as triangles, have recently been shown to divide at locations that can be predicted assuming a MinCDE-like reaction–diffusion mechanism for division site placement [205].

### Nucleoid-guided ParA-type ATPases

It becomes increasingly evident that nucleoid-bound ParA-type ATPases lead to pattern formation and cargo transport of proteins based on a similar mechanism as the MinCDE system. While these systems display a variety of distinct patterns, the underlying mechanism is similar. The ParA-type ATPase dimerizes upon ATP binding and is thereby enabled to associate with the matrix, in this case the nucleoid. The cognate ATPase-activating protein binds to, or is a structural part of, the cargo and stimulates the ATPase activity of the ParA-type ATPase upon contact. This interaction triggers the monomerization and release of the ParA-type ATPase from the DNA. How exactly the force for cargo transport is generated is still a matter of debate and several models have been proposed: (1) a filament-pulling mechanism [207, 208]; (2) a Brownian ratchet mechanism [209, 210]; (3) a DNA-relay mechanism [211]; and (4) a flux-based mechanism [212]. Independent of the exact mechanism, a variety of different nucleoid-guided ParA-type ATPases govern almost all aspects of spatiotemporal organization inside bacteria by forming distinct patterns. Most prominent are the plasmid and chromosome segregating ParABS systems that induce equal spacing of plasmids over the chromosome and partitioning of the daughter chromosomes during cell division, respectively. The ParA ATPases have been shown to be highly dynamic and oscillate over the nucleoid [213–215]. The cognate ATPase-activating protein is termed ParB and binds to the *parS* sites on the plasmids or chromosome. Next to segregation of nucleic components, ParA ATPases are also positive and negative regulators of FtsZ. For instance, the ParA-type ATPase MipZ from *C*. *crescentus* forms a gradient on the nucleoid that inhibits FtsZ polymerization [216]. The ParA-type ATPase PomZ from *Myxococcus xanthus*, in contrast, positions its cargo, a protein cluster formed by the two ATPase-activating proteins PomX and PomY, to midcell, which in turn stimulates divisome formation at midcell [212]. Furthermore, nucleoid-guided ParA ATPases also position other bacterial structures. For example, the ParA-type ATPase PpfA segregates chemotactic clusters in *Rhodobacter sphaeroides* [217]. Recently, it was shown that the ParA-type ATPase McdA and its cognate ATPase-activating protein McdB govern the equidistant positioning, and presumably the size and ultrastructure of carboxysomes in the cyanobacterium *S*. *elongatus* [218, 219].

### FlhG, a close relative of MinD

The ATPase FlhG, also termed YlxH, FleN, MotR or MinD2 [220] is closely related to MinD and belongs to the same subfamily of P-loop GTPases [30]. Like MinD, this protein relies on the membrane as a matrix. FlhG, together with the signal recognition particle GTPase FlhF, regulate the flagellation pattern in a variety of bacteria (see Schumacher et al. for a detailed review of the system [220]). For instance, FlhG/FlhF evenly distribute around 25 basal bodies over the cell surface in the peritrichously flagellated bacterium *B*. *subtilis* [220, 221] and ensure the localization of the single polar flagella in the monotrichous bacteria *Shewanella putrefaciens* and *Vibrio alginolyticus* [221, 222]. In the amphitrichous bacterium *Campylobacter jejuni*, FlhF/FlhG do not only regulate the two polar flagella, but deletion of FlhG also results in a minicell phenotype [223]. Hence, FlhG might serve as a regulator of cell division in this bacterium, too [223]. While FlhF seems to be mainly responsible for the correct localization of the flagella, FlhG appears to regulate the number of flagella. However, the reaction cycle of both proteins is intertwined: FlhG, unlike MinD, contains an N-terminal extension, termed activator helix, that directly interacts with FlhF activating its GTPase activity [224]. Like MinD, FlhG dimerizes in an ATP-dependent fashion allowing it to bind to the membrane via its C-terminal amphipathic helix [221]. Moreover, ATPase activity of FlhG was shown to be crucial for the correct flagellation pattern [222]. So far, it is unknown whether FlhG has a cognate ATPase-activating protein that could stimulate ATPase activity and its release from the membrane [221, 222].

The crystal structures of the MinD and FlhG dimers are highly similar [36, 221]. However, the monomeric structures differ: While the electron density of the MTS in the MinD monomer from *Pyrococcus furiosus* and *Archaeoglobus fulgidus* was absent [32, 33], the MTS is clearly visible in the structure of the monomeric FlhG [221]. Interestingly, in the FlhG monomer, the MTS is bound in a hydrophobic groove on top of the molecule. ATP-dependent dimerization leads to a conformational change that closes this hydrophobic groove, displaces the MTS, and enables it to bind to the membrane. Thus, in FlhG, the MTS is occluded in the monomer. In contrast, the missing electron density of the MinD MTS in the monomer structures indicates that it might always be solvent accessible in an open conformation. However, a monomer structure of the *E*. *coli* MinD has not been solved yet, thus not excluding the presence of such a structural rearrangement. For FlhG, the conformational switch of the MTS could be necessary to provide a more distinct difference between the ADP/ATP state in case the protein has no ATPase-activating protein, or simply due to the fact that the membrane-targeting sequence of FlhG is stronger than the MTS of *E*. *coli* MinD. Already a single copy of the FlhG MTS allows targeting of GFP to the inner membrane, whereas two copies of the *E*. *coli* MinD MTS are required for efficient binding [41, 221]. Future research will reveal more insights into this fascinating system, further highlighting similarities and differences to the MinCDE system.

## Harnessing the MinCDE system

The simplicity of the MinDE system and its nevertheless rich dynamics have made the Min system attractive for bottom–up synthetic biology. The MinCDE system will likely be one of the key components of a minimal division machinery in artificial cells.

Considerable work has been conducted to improve the control of MinCDE pattern formation. To this end, the geometry sensitivity of the system is harnessed on patterned bilayers or in custom-shaped microcompartments (see above) [80, 101]. Not only does the geometry control MinDE pattern formation, but also MinDE patterns can be seen as a biological sensor of cellular shape. More recently, the modification of a MinE peptide with a photoisomerizable crosslinker allowed optical manipulation of MinDE pattern formation in vitro [225]. With this photoswitch, MinDE patterns could not only be turned on and off, but also periodically entrained [225]. The ability to control patterns by light will not only allow to test mathematical models, but also has the potential to act as a biological hard drive storing spatial information in a biological system over several minutes.

Another key research goal is the spatiotemporal positioning of other molecules through MinCDE pattern formation. Reconstitution of MinCDE together with FtsZ-YFP-MTS in rod-shaped microcompartments demonstrated the capability of the system to position a simplified division machinery for the creation of a life-like entity [80]. Moreover, the recent discovery that MinDE in the absence of MinC can transport and position arbitrary membrane molecules even increases its applicability for the bottom–up construction of a minimal cell [42, 91]. Spatiotemporal positioning is crucial for such an endeavor, and the decoupling of the spatiotemporal positioning from the specific function of a protein, in this case MinC, is advantageous. To create artificial cells, molecular machineries of prokaryotic, eukaryotic or entirely synthetic origin such as DNA origami will be combined. Such machineries could now be positioned by MinDE patterns.

Another new mechanic aspect of MinDE oscillations was recently discovered through encapsulation of the system in giant unilamellar vesicles, leading to the shape deformation of the lipid interface and thus, a periodic beating or bouncing of the vesicles [88]. These results might facilitate the engineering of bioinspired “molecular robots” or active vesicles [88].

In recent years, the production of minicells has regained considerable interest [226]. The MinCDE system, which is conserved in many bacterial species, constitutes one of the best studied targets to achieve efficient minicell production. Minicells are employed for cryo-electron tomography, where their small size and lack of chromosomal DNA are advantageous in the visualization of molecular arrangements [226]. Additionally, minicells emerged as a powerful tool for the personalized cancer medicine, due to their ability to shuttle chemotherapeutics and selectively target cancer cells via bispecific antibodies [180, 181].

Hence, the MinCDE system is presently applied both, in vitro and in vivo. In vitro, the MinCDE system is controlled by geometric and optical cues and used to spatiotemporally position not only its native target FtsZ, but also other membrane-bound molecules, or to induce shape changes of free-standing membranes. In contrast, in vivo, so far not the action of the MinCDE system, but its total absence is exploited for the production of minicells.

## Conclusions

The compositional simplicity of the Min system, with only two proteins required for the formation of complex and oscillating patterns, makes it experimentally tractable and renders it suitable to be understood in depth. As such, MinDE have become a paradigm for pattern formation and for reaction–diffusion mechanisms in particular. With all tools in hands, in vivo observation and manipulation, in vitro reconstitution and in silico modelling, the field has thrived in the last years. By now, countless studies in vivo, in vitro and in silico deal with the MinCDE system, elucidating mechanistic details of the protein system itself, its coupling to pattern formation of downstream targets, or its applications. It further serves as an exquisite testbed to refine mathematical models by experimentalists and theoreticians teaming up.

The MinCDE system is closely related to nucleoid-guided ParA-type ATPases involved in every aspect of spatiotemporal organization in bacteria, and will hence allow to learn more about their mode of action, too. Furthermore, the system shows parallels to intracellular waves occurring in eukaryotes, that due to their compositional complexity remain enigmatic [161]. The ability of the MinDE system to spatiotemporally position other molecules through specific or non-specific interaction is appealing for the bottom–up construction of artificial cells.

Last but not least, it is also the mesmerizing dynamics of the MinDE patterns themselves that explains why the research on the MinCDE system has in the past years “kept many bacteriologists and biophysicists off the street” [124].
